# P2X-mediated AMPA receptor internalization and synaptic depression is controlled by two CaMKII phosphorylation sites on GluA1 in hippocampal neurons

**DOI:** 10.1038/srep31836

**Published:** 2016-09-14

**Authors:** Johan-Till Pougnet, Benjamin Compans, Audrey Martinez, Daniel Choquet, Eric Hosy, Eric Boué-Grabot

**Affiliations:** 1 Univ. de Bordeaux, Institut des Maladies Neurodégénératives, UMR 5293, F-33000 Bordeaux, France; 2 CNRS, Institut des Maladies Neurodégénératives, UMR 5293, F-33000 Bordeaux, France; 3 Univ. de Bordeaux, Institut Interdisciplinaire des Neurosciences, UMR 5297, F-33000 Bordeaux, France; 4 CNRS, Institut Interdisciplinaire des Neurosciences, UMR 5297, F-33000 Bordeaux, France; 5 Bordeaux Imaging Center, UMS 3420-US4 CNRS, INSERM, Université de Bordeaux, Bordeaux, France.

## Abstract

Plasticity at excitatory synapses can be induced either by synaptic release of glutamate or the release of gliotransmitters such as ATP. Recently, we showed that postsynaptic P2X2 receptors activated by ATP released from astrocytes downregulate synaptic AMPAR, providing a novel mechanism by which glial cells modulate synaptic activity. ATP- and lNMDA-induced depression in the CA1 region of the hippocampus are additive, suggesting distinct molecular pathways. AMPARs are homo-or hetero-tetramers composed of GluA1-A4. Here, we first show that P2X2-mediated AMPAR inhibition is dependent on the subunit composition of AMPAR. GluA3 homomers are insensitive and their presence in heteromers alters P2X-mediated inhibition. Using a mutational approach, we demonstrate that the two CaMKII phosphorylation sites S567 and S831 located in the cytoplasmic Loop1 and C-terminal tail of GluA1 subunits, respectively, are critical for P2X2-mediated AMPAR inhibition recorded from co-expressing *Xenopus* oocytes and removal of surface AMPAR at synapses of hippocampal neurons imaged by the super-resolution dSTORM technique. Finally, using phosphorylation site-specific antibodies, we show that P2X-induced depression in hippocampal slices produces a dephosphorylation of the GluA1 subunit at S567, contrary to NMDAR-mediated LTD. These findings indicate that GluA1 phosphorylation of S567 and S831 is critical for P2X2-mediated AMPAR internalization and ATP-driven synaptic depression.

The two major forms of synaptic plasticity in the brain - long term potentiation (LTP) and depression (LTD) - are thought to be involved in information storage and therefore in learning and memory as well as other physiological processes. The main forms of LTP and LTD triggered by either NMDAR or mGluR involve a long-lasting increase or decrease of synaptic strength, respectively resulting mainly from a rapid and long-lasting insertion or removal of AMPARs from the synapses^1^.

AMPARs are tetrameric complexes composed of GluA1-A4 subunits^2^. They form complexes with various associated proteins such as transmembrane AMPAR regulatory proteins (TARPs)^3^. These complexes are organized inside synapses by proteins of the post-synaptic density (PSD)^4^. The main AMPARs in the hippocampus are GluA1A2 and GluA2A3 heteromers as well as GluA1 homomers^1^,^5^. These AMPAR subunits have identified phosphorylation sites in their intracellular C-termini for several protein kinases that are bidirectionnally regulated during activity-dependent plasticity, with LTP increasing phosphorylation and LTD decreasing phosphorylation^4^,^6^,^7^.

Novel forms of plasticity at central synapses require the activation of astrocytes that drives the release of the gliotransmitter ATP and activation of extrasynaptic P2X receptors (P2X)^8^,^9^,^10^,^11^. Activation of astrocytic α1-adrenoceptors by noradrenaline (NA) or astrocytic mGluR by afferent activity induces astrocytic ATP release, providing mechanisms by which glial cells can respond to, and modulate synaptic activity^9^,^10^,^12^,^13^. The release of ATP by astrocytes causes a long-lasting increase of glutamatergic synaptic currents in magnocellular neurons, scaling glutamate synapses in a multiplicative manner in the paraventricular nucleus of the hypothalamus. In this case, ATP activates postsynaptic P2X7 which promotes the insertion of AMPAR through a phosphatidylinositol 3-kinase (PI3K)-dependent mechanism^8^,^9^. However, P2X7 is restricted to specific neuronal populations^14^ while P2X2 and P2X4 are widely expressed in the brain^15^. Recently, we showed that an activation of postsynaptic P2X2 by astrocytic release of ATP causes an enduring decrease of postsynaptic AMPAR currents in hippocampal neurons and a depression of field potentials recorded in the CA1 region of mouse brain slices^10^. Ca^2+^ entry through the opening of P2X2 channels triggers internalization of AMPARs, leading to reduced surface AMPARs in dendrites and at synapses^10^. Such a depression of AMPA current and surface GluA1 or GluA1A2 numbers can be reproduced in a heterologous system (*Xenopus* oocytes) following activation of co-expressed P2X2. In addition, NMDA- and ATP-dependent depression are additive in CA1 neurons indicating that P2X- and NMDAR-dependent internalization of AMPAR use distinct signaling pathways^10^. Indeed, P2X-driven synaptic depression and inhibition of AMPAR in oocytes are abolished by a blockade of phosphatase or CaMKII activities, while calcineurin, PKA or PKC inhibitors have no effect^10^. This contrasts with the conventional NMDAR-dependent plasticity model where phosphorylation by CaMKII kinase is associated with LTP and dephosphorylation by calcineurin of AMPAR is required for LTD^4^,^16^. and suggests that during P2X2 activation a novel form of regulation of AMPAR subunits occurs.

Here, we show that P2X2-mediated AMPAR inhibition is GluA1 or GluA2 subunit specific. We further investigated the differential structural requirement of GluA1 and have identified two critical residues, S831 and S567 phosphorylated by CaMKII, that are crucial for P2X2-mediated inhibition and the removal of surface GluA1-containing AMPAR at the synapses. Finally, we show that S567 of GluA1 is dephosphorylated during P2X-mediated LTD in the hippocampus while no change occurs at S831 and S845, two crucial sites for NMDAR-dependent plasticity^6^,^16^,^17^.

## Results

### P2X2-mediated AMPAR inhibition is dependent on GluA subunits

**Figure 1:**
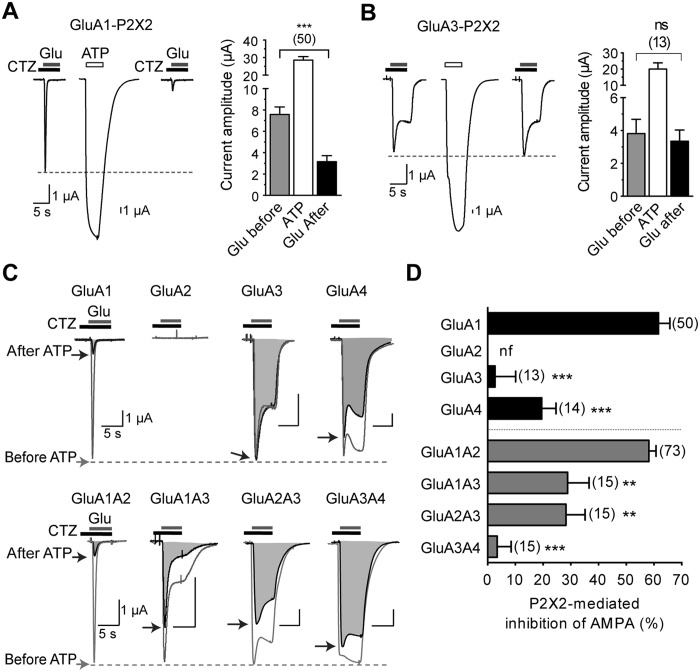
P2X2-mediated inhibition of AMPAR current is dependent upon AMPAR subunit composition. (**A,B)** Representative currents evoked by application of glutamate (Glu 1 mM for 5 s) in the presence of cyclothiazide (CTZ, 100 μM, 10 s of preincubation) before and 2 min after an ATP-induced current (100 μM) in oocytes co-expressing P2X2 and either GluA1 (A) or GluA3 subunits. (**B)** Summary of amplitude averages of AMPAR and P2X2 currents. (**C)** Superimposed AMPAR currents evoked in the same conditions as in A,B before (gray traces, unfilled areas) and 2 min after an ATP-induced current (black traces and shaded areas) for oocytes expressing P2X2R and indicated homomeric or heteromeric AMPARs. (**D)** Bar graphs summarizing the extent of inhibition (expressed as %) of homomeric or heteromeric AMPAR after activation of P2X2R. Statistical differences compared to GluA1 or GluA1A2 are indicated. **P < 0.01; ***P < 0.001; ns, P > 0.05; Error bars represent s.e.m.; Numbers of cells are indicated in parentheses. nf, non-functional.

We previously showed that P2X2 activation triggers a dynamin-dependent internalization of homomeric GluA1 or heteromeric GluA1A2 AMPAR, leading to reduced surface AMPAR density and current both in neurons and a recombinant expression system^10^. To evaluate the impact of P2X2 activation on AMPARs, we first examined changes of AMPAR current following P2X2 activation using two electrode voltage clamp recordings from *Xenopus* oocytes co-expressing P2X2 and each GluA1-4 subunit alone or in pair-wise combination (Fig. 1). AMPAR responses were evoked by application of glutamate (Glu 1 mM, 5 s) in the presence of cyclothiazide (CTZ 100 μM, 10 s of preincubation), a blocker of AMPAR desensitization to ensure detection of the whole AMPAR current. Two minutes after a single ATP-evoked current (ATP 100 μM, 5 s), the amplitude of homomeric GluA1 current was drastically reduced from 7.58 ± 0.70 μA (before) to 3.17 ± 0.54 μA (after) (P < 0.001, n = 50, Fig. 1A,D) as previously described^10^. In contrast to GluA1 receptor inhibition, ATP-evoked P2X2 current did not change homomeric GluA3 responses (3.81 ± 0.87 μA (before), 3.35 ± 0.67 μA (after), P > 0.05, n = 13, Fig. 1B–D). We superimposed the AMPAR current recorded before and after the ATP-evoked current to visualize directly the P2X-mediated inhibition of AMPAR (Fig. 1C,D). In contrast to the drastic inhibition of GluA1 (by 61.64 ± 4.11%), an inhibitory effect of P2X2 activation on homomeric GluA3 receptors was virtually absent, with the measured residual inhibition of GluA3 (2.71 ± 7.39%, Fig. 1C,D) being non-significantly different from zero (P > 0.05, n = 13). However, GluA4 receptors were decreased following P2X2 activation (19.53 ± 5.15% of inhibition, n = 14). Although this inhibitory effect was significantly smaller (P < 0.001) than that observed with GluA1 receptors, it remained significantly different from zero inhibition (P < 0.05). P2X-mediated inhibition of GluA2 could not be tested since GluA2 subunits did not form functional homomeric receptors. Since native AMPAR are predominantly heteromers, it was also important to determine the effect of P2X activation on heteromeric AMPAR. As shown in Fig. 1C and as previously reported^10^, the inhibition of heteromeric GluA1A2 was similar to that of homomeric GluA1 (58.17 ± 2.57%, n = 73). In contrast, GluA1A3 and GluA2A3 showed significantly less P2X-mediated inhibition (28.87 ± 7.72% and 28.25 ± 6.92% respectively, n = 15 for each) and GluA3A4, like homomeric GluA3, displayed a negligible inhibitory influence (3.44 ± 7.39%, n = 15) that was not significantly different from zero (P > 0.05). These results thus indicated that P2X2-mediated alteration of AMPAR function and trafficking is a subunit-specific mechanism. GluA3 subunits are insensitive to P2X2 activation and their presence in the receptor complexes alters P2X-inhibition of heteromeric GluA1 or GluA2-containing receptors.

### Residues of the CT of GluA1 contribute to the P2X-mediated AMPAR inhibition

**Figure 2:**
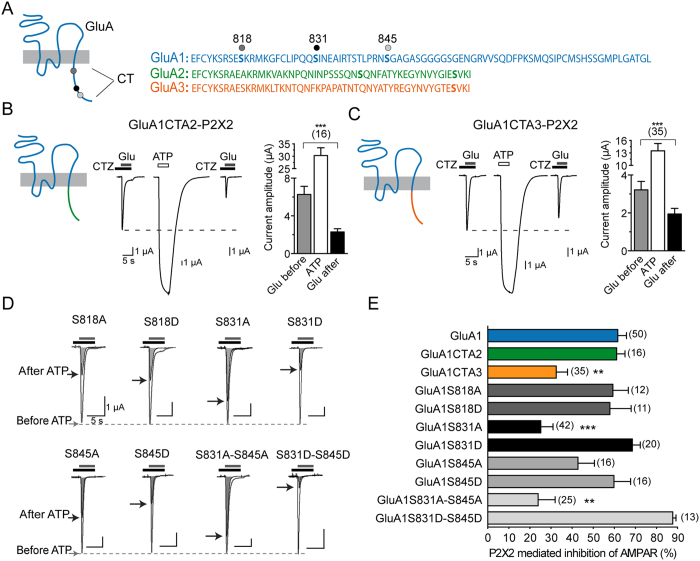
The carboxy tail of the GluA1 and Ser831 residue is necessary but not sufficient for P2X-mediated AMPAR depression. (**A**) Schematic of AMPAR subunit topology and sequence alignment of the intracellular carboxy-terminal tails (CT) of GluA1-A3 subunits. The three main phosphorylation sites on GluA1 known to contribute to synaptic plasticity are indicated by dots. **(B,C)** Chimeric GluA1 receptors with the intracellular CT of either GluA2 (**B**) or GluA3 (C) subunits were designed to determine the region involved in the inhibitory effect of P2X2 activation. Representative currents evoked by applications of glutamate (Glu 1 mM, 5 s) in the presence of cyclothiazide (CTZ, 100 μM, 10 s of preincubation) before and 2 min after an ATP-induced current (100 μM) in oocytes co-expressing P2X2 and chimeric GluA1CTA1 or GluA1CTA3 receptors. The mean amplitudes of currents are also indicated. (**D)** Superimposed glutamate-evoked currents before and after ATP-induced P2X2R current recorded in the same conditions as in (**B**) for point or double GluA1 mutants. Ser818, Ser831 and Ser845 were mutated into alanine (**A**) or phosphomimetic aspartate residues (**D**). Maximal amplitude after ATP-induce currents are indicated by black arrows. (**E)** Summary bar graph representing the percentage of P2X2-mediated AMPAR current inhibition for wild-type, chimeric and mutated GluA1 receptors. Statistical differences compared to GluA1 are indicated. **p < 0.01; ***p < 0.001 number of cells is indicated between brackets.

GluA subunits display sequence divergence within the C-terminal cytoplasmic domains that have been shown to contain phosphorylation and/or protein interaction sites controlling membrane trafficking (Fig. 2A)^1^. To identify whether AMPA subunit C-terminal domains (CT) confer the subunit specificity of the P2X-mediated inhibition of AMPAR, we first constructed chimeric GluA1 subunits in oocytes in which the CT had been swapped with either GluA2 or GluA3 CT and compared the P2X2-induced AMPAR inhibition (Fig. 2A–C). GluA1CTA2 were inhibited by P2X2 to the same extent as GluA1 (61.08 ± 3.99% inhibition, n = 16, Fig. 2B,E). In contrast, GluA1CTA3 were partially inhibited following P2X2 activation. Thus, replacing the CT of GluA1 with that of the P2X2-insensitive GluA3 subunits significantly reduced the inhibition to 32.49 ± 5.34%, P < 0.01, n = 35, Fig. 2C,E). This set of experiments therefore indicates that the CT of GluA1 as well as that of GluA2 subunits contributes to the P2X-mediated inhibition of AMPAR, albeit only partially. Since GluA1, in contrast to GluA2, is sufficient to mediate P2X-induced inhibition and form functional homomers, we next focused attention on the structural requirement of GluA1 for this effect. We first tested whether the phosphorylation sites S818, S831 and S845 within the CT of GluA1 are implicated in the P2X2-mediated inhibition by co-expressing mutants of GluA1 with P2X2 in oocytes. Replacement of S818 by an alanine (S818A) or phosphomimetic aspartate (S818D) did not modify the extent of P2X-mediated inhibition of GluA1 (59.30 ± 7.34%, n = 12 and 57.90 ± 10.08%, n = 13 respectively, Fig. 2D,E). A similar inhibition of GluA1S845A or S845D was also observed (Fig. 2D,E). Interestingly, the extent of P2X-mediated inhibition of GluA1S831A was significantly smaller compared to that of GluA1 (25.14 ± 5.80%, p < 0.01, n = 42) and was similar to the inhibition observed for GluA1CTA3 (Fig. 2C,E). Such a reduction in P2X-mediated inhibition was not expressed by the phosphomimetic mutation S831D (GluA1S831D inhibition was 68.57 ± 3.74%, n = 20). Double mutants GluA1S831AS845A or GluA1S831DS845D exhibited the same P2X-mediated change as the single mutant S831A or S831D, respectively. Together these results point to the S831 residue of the CT as a target for the P2X-mediated regulation of GluA1 receptor trafficking and function, but also suggest that regions other the CT contribute to these processes.

### Identification of key residues in the loop1 and the C-tail of GluA1 mediating the P2X2-mediated AMPAR inhibition

**Figure 3:**
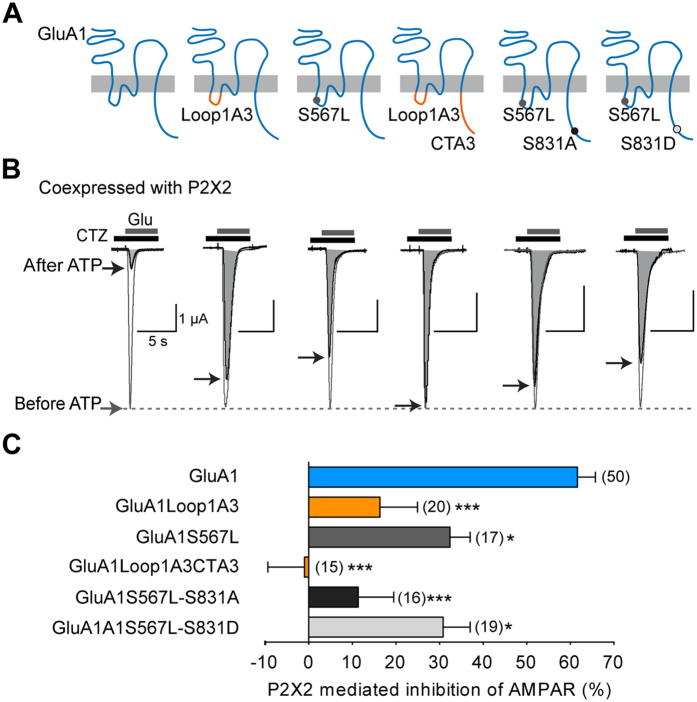
S567 and S831 residues of GluA1 are involved in the P2X2-mediated AMPAR current inhibition. (**A**) Schematic representation of chimeric or mutant GluA1 receptors of the first intracellular loop and CT of GluA1. (**B)** Surimposed glutamate-evoked currents before and after ATP-induced P2X2R current recorded in the same conditions described in Fig. 2 for oocytes co-expressing P2X2R and the corresponding modified GluA1 subunits. Maximal amplitudes of AMPAR currents after ATP (shaded areas) are indicated by black arrows. (**C)** Bar graphs showing the percentage of P2X2-mediated AMPAR current inhibition for wild-type GluA1 and chimeric or mutated GluA1 receptors. Statistical differences compared to GluA1 are indicated. *p < 0.05; ***p < 0.001; Numbers of cells are indicated in parentheses.

We thus explored the possibility that the intracellular loop1 of GluA1 that is critical for AMPAR trafficking^18^ plays a role in the P2X-mediated internalization of GluA1-containing receptors. We first compared the P2X-induced inhibition of glutamate-evoked responses of GluA1 to that of GluA1loop1A3 in which the loop1 of GluA1 was swapped with the loop1 of P2X-insensitive GluA3 subunits (Fig. 3A–C). The P2X-mediated inhibition of GluA1loop1A3 (16.34 ± 8.64%, n = 35, Fig. 3A–C) was abolished (i.e., became insignificantly different from zero inhibition) indicating that GluA1loop1A3 currents were not inhibited by ATP currents. S567 is a critical CaMKII site that regulates loop1-dependent AMPAR trafficking as shown by experiments using a non-phosphorylatable S567L mutant^18^,^19^. We thus examined the implication of S567 by co-expressing the S567L mutant with P2X2 and observed a significant decrease in P2X2-induced inhibition of GluA1S567L (29.83 ± 4.43%, n = 17). However, the inhibition was not abolished (P < 0.05 compared to zero inhibition). This finding therefore shows that loop1 and S567 of GluA1 contributes to the P2X-mediated regulation of AMPAR trafficking. Finally, we replaced both the intracellular loop1 and CT of GluA1 by swapping both domains with the one of the GluA3 subunits. In co-expressing cells, glutamate-evoked current of the GluA1Loop1A3CTA3 were not modified by P2X2 activation (−0.97 ± 8.38% of inhibition, n = 15). P2X2-induced inhibition was also almost abolished with the double mutant GluA1S567L-S831A (11.36 ± 8.13% of inhibition, n = 16, not significantly different from zero inhibition), while the extent of GluA1S567LS831D inhibition (30.81 ± 6.21%, n = 19) was similar to that of the single mutant GluA1S567L (Fig. 3A–C), suggesting that both the S567 and S831 residues play a prominent role in the bP2X-induced inhibition of AMPAR.

**Figure 4:**
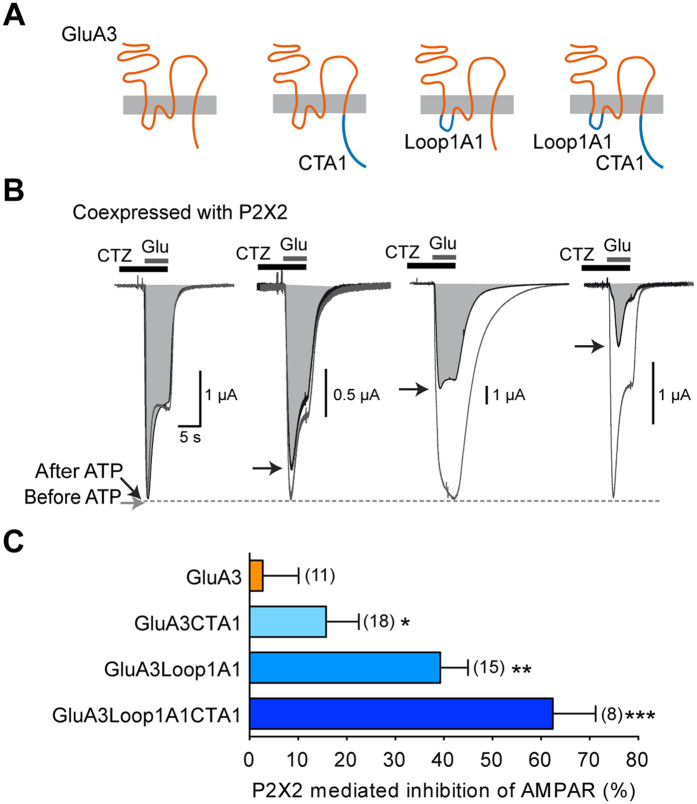
Chimeric GluA3 subunits with intracellular loop1 and CT of GluA1 confers P2X2-mediated inhibition on GluA3 receptors. (**A**) Schematic representation of chimeric GluA3 subunits bearing the first intracellular loop and/or the CT of GluA1. (**B)** Surimposed glutamate-evoked currents before and after ATP-induced P2X2R currents recorded from oocytes co-expressing P2X2R and the corresponding modified GluA3 subunits. Maximal amplitude of AMPAR currents after ATP (shaded areas) are indicated by black arrows. (**C)** Bar graph showing the percentage of P2X2-mediated AMPAR current inhibition for wild-type GluA3 and chimeric GluA3 receptors. Statistical differences compared to GluA3 are indicated. *p < 0.05; ***p < 0.001; Numbers of cells are indicated in parentheses.

Could the intracellular domains of GluA1 confer P2X2-mediated inhibition on the insensitive GluA3 receptors? To answer this question, we performed reciprocal experiments by swapping the intracellular loop1 and/or the CT of GluA3 by the homologous domains of GluA1 and measured the effect of P2X activation on glutamate-evoked responses in these constructs (Fig. 4). GluA3CTA1 responses were not significantly inhibited following P2X2 activation. The percentage of inhibition (15.74 ± 6.78%, n = 18) was significantly different (P < 0.05) from that of GluA3, but not from zero inhibition (P > 0.05). GluA3loop1A1 was partially but significantly inhibited after P2X2 activation (39.28 ± 5.69% n = 15). Interestingly, GluA3loop1A1CTA1 fully rescued the P2X mediated inhibition that was absent for GluA3 (62.43 ± 8.78%, n = 8), to the same extent as GluA1 receptors, indicating that both domains contribute to the P2X inhibition (Fig. 4B,C). We also mutated the L577 residue of GluA3 into a serine, the residue equivalent to S567 of GluA1. However, GluA3L577S were almost insensitive to P2X2 activation (not shown) as for GluA3, suggesting that a reintroduction of the single serine residue in loop1 is insufficient to rescue the site required for the P2X-mediated inhibition of GluA1.

Together these data show that S567 and S831 are critical residues for the P2X-dependent GluA1-containing AMPAR alteration, supporting the idea that P2X2 induced changes of the phosphorylation state of GluA1 subunits.

### GluA1 S831 and S567 residues are crucial for P2X-mediated internalization in hippocampal neurons

**Figure 5:**
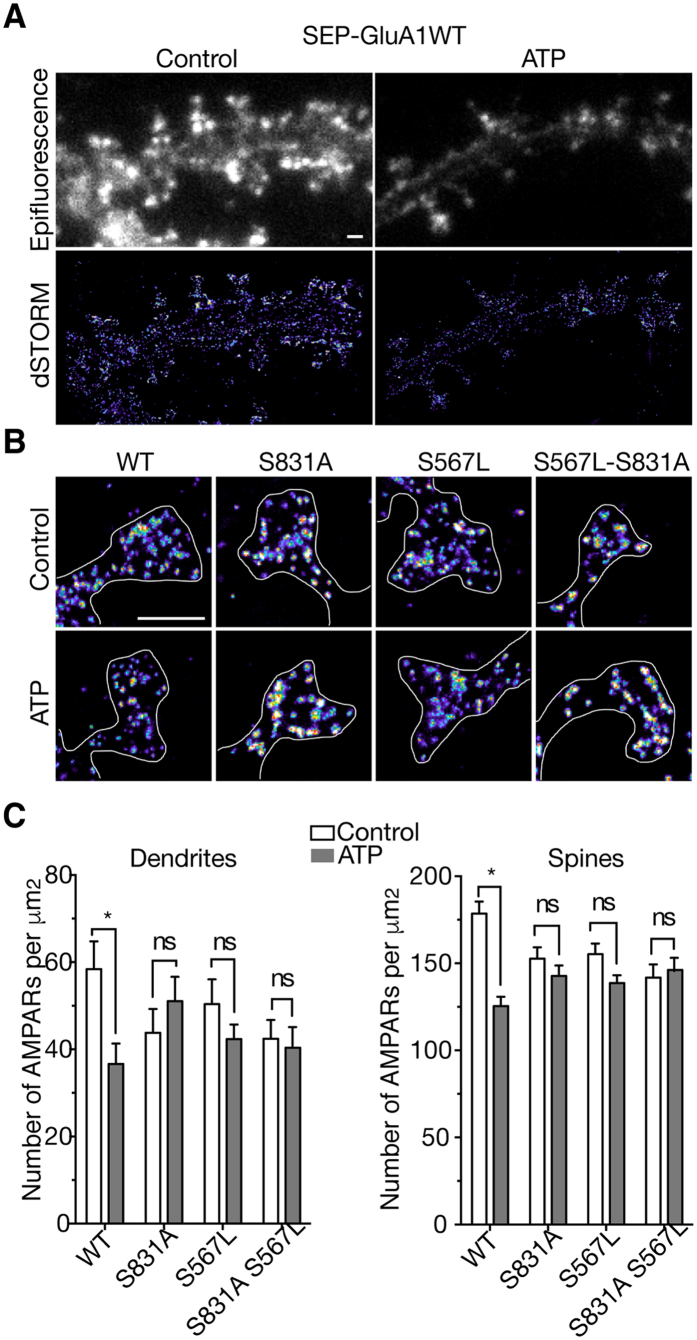
Decrease in number of dendritic and synaptic SEP-tagged GluA1 receptors triggered by activation of native P2XR in transfected hippocampal neurons is mediated by the S831 or S567 GluA1 residues. (**A**) Epifluorescence (upper panels) and super-resolution images (bottom panels) reconstructed from direct Stochastic Optical Reconstruction Microscopy (dSTORM) of wild-type (WT) SEP-tagged GluA1 in transfected hippocampal neurons labeled with surface anti-GFP antibodies before (control, left panel) and after ATP treatments (right panel). (**B**) Representative dSTORM images of spines from neurons expressing wild-type SEP-tagged GluA1 and mutant GluA1 S831A, S567L and double mutant in control conditions or 1a0 min after application of ATP (100 μM, 1 min) in presence of CGS15943 (3 μM) and TTX (0.5 μM). Scale bars, 1 μm. (**C**) Average density values of wild-type and mutant SEP-GluA1-containing AMPAR in synapses and dendrites in control condition (unshaded bars) and after P2XR activation (shaded bars). *P < 0.05; ns, P > 0.05; Error bars: s.e.m.

We next examined whether the identified molecular determinants of GluA1 subunits are crucial for an P2X2-mediated removal of surface GluA1-containing AMPAR in hippocampal neurons. Using dSTORM super-resolution imaging, we previously showed that an activation of endogenous P2X receptors reduces the density of native GluA2-containing receptors at the surface of hippocampal dendrites and synapses^10^. Using the same dSTORM imaging technique, we first measured the density of AMPAR on rat hippocampal neurons transfected with super ecliptic pHluorin (SEP)-tagged GluA1 and stained with anti-GFP antibodies coupled to Alexa 647 (Fig. 5A,C). SEP-GluA1 containing AMPARs are clustered at synapses, with a higher number of receptors (178.5 ± 6.9/μm^2^, n = 67) than the dendrites (58.43 ± 6.37/μm^2^, n = 13) as previously shown for endogenous AMPARs^10^,^20^. Ten minutes after activation of endogenous P2X receptors using 100 μM ATP in the presence of tetrodotoxin (TTX 0.5 μM) and the adenosine receptor antagonist CGS15943 (3 μM), we observed a significant reduction in surface SEP-GluA1 containing receptors in spines and dendrites (after ATP: 125.5 ± 5.3/μm^2^, n = 73 and 36.63 ± 4.69/μm^2^, n = 14, respectively, Fig. 5A–C) showing that activation of native P2X receptors triggers an internalization of GluA1-containing AMPAR as previously observed for endogenous GluA2-containing receptors^10^. In transfected hippocampal neurons, mutant GluA1S831A, S567L or double mutant S567LS831A exhibited a similar density of AMPAR in both dendrites and spines in control conditions compared to the SEP-GluA1 wild-type. This indicates that these mutations did not significantly alter surface AMPAR trafficking or clustering in spines in basal conditions even though a recurrent small decrease of total AMPAR number could be observed (Fig. 5B,C). However, ATP did not trigger any significant changes in AMPAR numbers in the dendrites and spines of single or double mutants, confirming that both residues are important for P2X-mediated AMPAR internalization in hippocampal neurons.

### GluA1 S567 is dephosphorylated during P2X-driven LTD in the hippocampus

**Figure 6:**
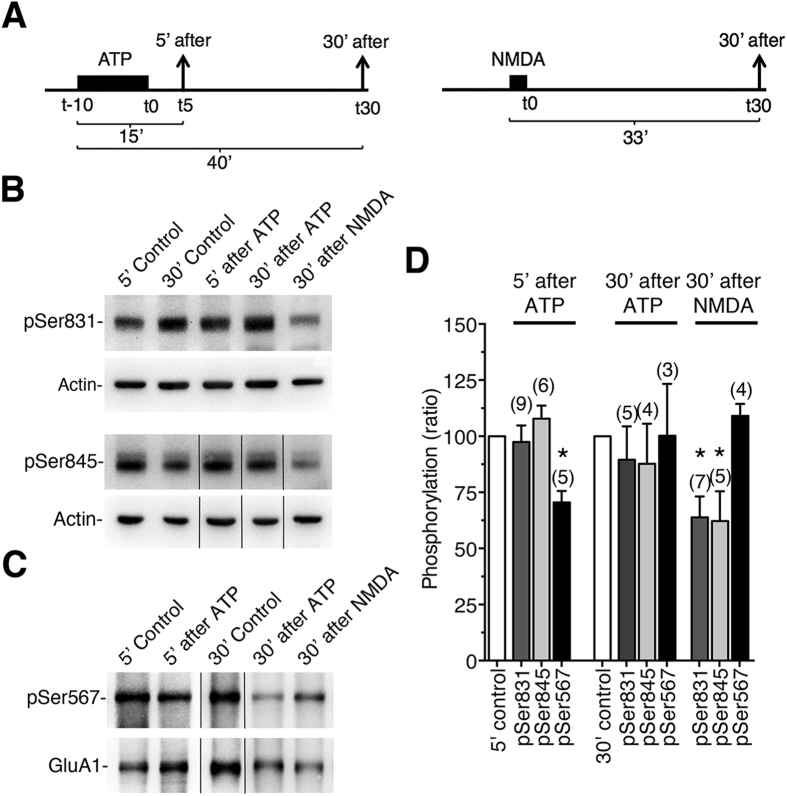
Dephosphorylation of the S567 site of the AMPAR GluA1 subunit during P2XR-mediated synaptic depression in hippocampal slices. (**A**) Experimental design of ATP or NMDA- induced synaptic depression in hippocampal slices. (**B**) Crude membrane fractions from control hippocampal slices and ATP-induced synaptic depression or NMDAR-induced LTD slices taken at indicated times (5′ and/or 30′) after the application of ATP (300 μM for 10 min) or NMDA (20 μM for 3 min) respectively, separated under SDS-PAGE and blotted using antibodies against phospho-S831, phospho-S845 and actin. (**C**) Crude membrane fractions from hippocampal slices treated as described in A, immunoprecipated using anti-GluA1 before separation under SDS-PAGE and blotted using antibodies against anti-phospho S567 and anti-GluA1. Cropped blots are displayed (see Supplementary Fig. 1.) (**D**) Quantification of the relative amounts of phosphorylation of GluA1 on S831, S845 and S567 at indicated time points after ATP or NMDA treatments. Bars represent the ratio of the signals for the anti-phospho site specific antibodies and the actin or the anti-GluA1 normalized to control at each time point. The number of independent experiments is indicated in parentheses. *P < 0.05.

We previously showed that perfusion of ATP (300 μM) on acute hippocampal slices from 4–5 week-old mice in the presence of blockers of adenosine and NMDA receptors, consistently produced a long-lasting depression of field excitatory post-synaptic potentials, which was in turn blocked by P2X antagonists^10^. We next sought to determine if the P2X-mediated depression in the hippocampus is associated with a change in the phosphorylation of GluA1 using antibodies against the phosphorylation site-specific of GluA1 subunits. Crude membrane proteins isolated from hippocampal slices at different time points (Fig. 6A) after ATP treatment (300 μM, 10 min) or without treatment were immunoblotted using antibodies against phosphoSer-831, phosphoSer-845 and phosphoSer-567 antibodies (Fig. 6B and Supplementary Fig. 1). For the detection of phosphoSer-567, an immunoprecipitation step using anti-GluA1 antibodies was necessary prior to immunoblotting (see Methods and Supplementary Fig. 2). NMDA treatment (20 μM, 3 min) was also applied to hippocampal slices to trigger chemical LTD and to determine whether P2X- and NMDA- dependent LTDs involved distinct changes in GluA1 AMPAR (Fig. 6A). Western blotting confirmed that S831, S845 and S567 have a significant level of basal phosphorylation in hippocampal slices (control conditions, Fig. 6B–D)^16^,^19^ and demonstrated that the phosphorylation of Serine 831 or Serine 845 remained unchanged during P2X-mediated LTD. Interestingly, phosphorylation of Serine 567 detected by using anti-phosphoS567 was significantly reduced (70.49 ± 5.1% of control, P < 0.05, n = 5) 5 minutes after ATP treatment (i.e., 15 min after the onset of ATP application, Fig. 6). The dephosphorylation of S567 was not observed 30 min after ATP treatment (i.e., 40 min after ATP application onset), as for the P2X-mediated LTD^10^. In contrast, phosphorylation of S831 and S845 was significantly reduced (63.85 ± 9.25% and 62.18 ± 13.32% of control, respectively; P < 0.05, n = 5–7) 30 min after NMDA treatment as expected^6^,^16^. Phosphorylation of S567 after NMDA treatment showed a tendency to increase (109.1 ± 5.31% of control, n = 4) that was smaller than the increase of phospho-S567 reported during low frequency stimuli-induced LTD^19^. Thus the dephosphorylation of the S567 CaMKII site of GluA1 after ATP treatment strongly correlated with the P2X-mediated depression of synaptic responses both in terms of time course and the blockade by the CaMKII inhibitor KN-93^10^.

## Discussion

Using a mutational approach, we show here that the two CaMKII phosphorylation sites S567 and S831 located respectively in the cytoplasmic domain Loop1 and the CT of GluA1 subunits are critical for the P2X-mediated internalization and current inhibition of GluA1-containing AMPAR. Our results also suggest that phosphorylation levels of both S567 and S831 is important for P2X-driven depression in the hippocampus. Previously, we described a P2X2-mediated internalization of AMPAR that is critical for the prolonged decrease of hippocampal synaptic strength triggered by an astrocytic release of ATP^10^. Similarly to an NMDA-induced AMPAR internalization^21^, the activation of P2X2 or P2X4 triggers AMPAR internalization and AMPAR current inhibition in hippocampal neurons as well as in an heterologous system, and this trafficking event requires primarily a Ca^2+^ influx through the direct opening of P2X2 channels^10^. Because NMDAR- and P2X-dependent synaptic depression are not occlusive in the hippocampus and since P2X-mediated AMPAR internalization and synaptic depression requires, both *in situ* and *in vitro*, phosphatase and CaMKII activities in contrast to NMDAR-dependent internalization and LTD^16^,^22^, we aimed here to explore the signaling mechanism that triggers P2X-induced AMPAR internalization and current inhibition by focusing primarily on the nature of AMPAR itself. We found by co-expressing P2X2 and each GluA1–4 subunit alone or in two subunit combinations in *Xenopus* oocytes that a P2X2-mediated alteration of AMPAR is dependent on the latter’s composition. In contrast to homomeric GluA1 or heteromeric GluA1A2 that were strongly inhibited (~60%) (Fig. 1, see also^10^), the inhibition of homomeric GluA4 was less pronounced (~20%) and homomeric GluA3 receptors were completely insensitive to P2X2 activation. In addition, the presence of GluA3 in the receptor complexes significantly reduced the P2X-inhibition of the heteromers GluA1A3 or GluA2A3, indicating a negative impact of GluA3 on the ATP-induced inhibition. These results therefore show the pivotal role played by GluA1 or GluA2 in the P2X-mediated AMPAR alteration and also indicate that P2X may regulate to a varying extent the function and trafficking of the main AMPARs in the hippocampus, which are GluA1A2 or GluA2A3 as well as GluA1 homomers^1^,^4^,^5^,^23^. GluA1–4 subunits have similar extracellular N-terminals and transmembrane domains but differ significantly in their C-terminal (CT) cytoplasmic tail regions which contain multiple regulatory elements that are subject to phosphorylation and/or interaction with scaffold proteins. These latter proteins play a crucial role in the regulation of AMPAR function, trafficking, lateral mobility and synaptic plasticity^4^,^24^. GluA2 was considered as the primary determinant during NMDAR-dependent internalization of synaptic AMPARs and LTD through modifications of the CT of GluA2 and interaction with scaffolding proteins^4^. However the fact that LTD occurs normally in mice lacking GluA2 and GluA3 in the hippocampus indicated that GluA2 is not essential for hippocampal LTD^25^. Other studies suggested that LTD and AMPAR internalization require calcineurin and dephosphorylation of PSD proteins and/or PKA and PKC sites within the CT of GluA1. In addition knock-in mice containing mutations in the GluA1 CaMKII and PKA phosphorylation sites display significant deficits in expressing LTD^7^,^16^,^26^,^27^,^28^,^29^. To test whether the signal that confers subunit specificity of P2X-mediated alteration resides within the CT of GluA subunits, we swapped the CT of GluA1 with the CT of GluA2 or GluA3 (Fig. 2). Chimeric receptors showed that replacing the CT of GluA1 with that of GluA2 did not modify the P2X2-induced inhibition of AMPAR. The replacement of GluA1CT with the GluA3CT significantly reduced, but did not abolish, the inhibition. These results therefore indicate that the CT of GluA1 or GluA2 subunits participate, but that regions other than the CT of the AMPAR subunit may also contribute, in the P2X-mediated inhibition of AMPAR. Since GluA2 homomers do not form functional receptors, we focused on GluA1 subunits to identify the molecular determinants of the P2X-mediated inhibition. Interestingly, by single or double mutation of the three serine located within the CT of GluA1 and described as the phosphorylation substrates for alanine or phosphomimetic aspartate (S818, S831 and S845; phosphorylation sites for PKC, PKC/CaMKII and PKA, respectively)^30^ we showed that only the substitution of S831 by an alanine significantly reduced the P2X-mediated inhibition. Moreover, this reduction in inhibition was similar to that observed with chimeric GluA1CTA3 receptors. These results excluded a contribution of S818 or S845 in agreement with the previously reported pharmacological insensitivity of the P2X-driven depression to blockade of PKA or PKC activities^10^. In addition, S831D was fully inhibited during P2X2 activation indicating that phosphorylation of S831 is a prerequisite, but not alone sufficient, to produce the observed P2X-mediated AMPAR alteration.

The first intracellular loop1 of GluA1, which was also shown to be critical for AMPAR trafficking and targeting to the synapse, contains a CaMKII site (S567) that is phosphorylated both *in vitro* and *in vivo*^18^, and recent work has suggested that CaMKII activities is also required for both hippocampal LTP and LTD^19^. By swapping the Loop1 of GluA1 by the homologous domain of the P2X-insensitive GluA3 subunits (Fig. 3) we showed that such a loop 1 replacement almost abolished the P2X2-mediated inhibition, with the remaining inhibition (~15%) being insignificantly different from zero. By replacing both loop1 and the CT of GluA1 by the homologous domain of GluA3 or by mutating the two CaMKII sites by nonphosphorylatable residues S567L and S831A, the P2X2-mediated inhibition was completely abolished. Conversely, by replacing both the loop1 and the CT of GluA3 by the homologous domain of GluA1, we fully rescued the P2X-mediated inhibition (~60%) of the insensitive-homomeric GluA3 receptors, thereby confirming that both CaMKII sites located in the intracellular loop1 and CT of the GluA1 subunit are critical for P2X2-mediated inhibition of AMPAR arising from an alteration in the number of surface AMPARs^10^. These data were reinforced with imaging experiments on hippocampal neurons transfected with wild-type or mutant GluA1. Specifically, the use of super-resolution dSTORM imaging showed that activation by ATP of endogenous P2X receptors caused the loss of wild-type SEP-tagged GluA1-containing AMPAR from synapses and dendrites in hippocampal neurons as previously observed for native GluA2-containing receptors^10^. Importantly, as expected, no change in the number of mutant GluA1 following ATP activation of endogenous P2X was observed in hippocampal neurons transfected with the double mutant GluA1S567L-S831A. More surprisingly, the ATP-driven internalization was also completely abolished for both single mutant GluA1S567L and GluS831A. We noted a tendency towards a decrease of total AMPAR number in neurons transfected with mutants compared to wild-type GluA1 but neither S831 nor S567 mutation significantly altered the synaptic targeting of AMPAR in basal conditions, i.e., in the absence of synaptic activity. This is consistent with previous studies showing that the GluA1 CT plays a role in surface delivery of GluA1 and synaptic plasticity and but not in basal synaptic transmission or synaptic trafficking^6^,^31^,^32^. Our super-resolution imaging experiments on the S567L mutant contrast with previous colocalization experiments of GluA1 and PSD-95 indicating that GluA1 S567 contributes to the synaptic targeting of AMPAR^18^. These latter experiments were performed by molecular replacement, i.e., expression of GluA1S567 mutant on an AMPAR null background which allowed the specific role of S567 in AMPAR synaptic localization to be defined^18^. This discrepancy may be easily explained but the fact that here we expressed mutant GluA1S567L on a wildtype background, leaving open the possibility that these subunits form complexes with native subunits and/or associated proteins that may ensure proper synaptic localization. These compensatory effects may also explain the differences in the magnitude of the effects on both single mutant GluA1S567L or GluA1S831A observed between the two expression systems. P2X2-mediated inhibition of both single mutants was partially reduced in oocytes, whereas their respective internalizations were fully suppressed in transfected neurons. The association with native wild-type AMPAR subunits such as GluA3 could facilitate P2X-mediated internalization in transfected neurons. In addition, our experiments using phosphorylation site-specific antibodies showed that ATP-induced synaptic depression from hippocampal slices^10^ is associated with a fast and reversible dephosphorylation of GluA1 at the CaMKII phosphorylation site S567 without change in the phosphorylation levels of S845 or S831. Furthermore, the kinetics of dephosphorylation were consistent with the duration of the synaptic depression observed by field recordings from hippocampal slices^10^. NMDAR-dependent LTD revealed opposite results with no change in S567 and dephosphorylation of S845 and S831. Although a recent study reported a low level of basal phosphorylation of GluA1 subunits, indicating that the phosphorylation changes of GluA1 during synaptic plasticity may require alternative interpretations^33^, NMDAR-dependent or low frequency stimulation (LFS)-induced LTD associated with a persistent dephosphorylation of S845 and a transient dephosphorylation of S831 has been frequently observed^1^,^7^,^16^,^22^,^26^,^28^.

Together our findings provide evidence that the ATP-mediated internalization of GluA1-containing AMPAR requires two sites, S567 and S831, of GluA1 subunits and that dephosphorylation of GluA1 may contribute to the depression of synaptic activity in the hippocampus. We previously showed that P2X activation depressed naïve synapses as well as synapses already depressed by LFS in the CA1 region^10^. Since high frequency stimulation (HFS)-induced LTP increases phosphorylation of S831^1^,^6^, it will be important to now determine whether P2X activation may dephosphorylate S831 during LTP and thereby contribute to synaptic depotentiation.

## Materials and Methods

### Constructs

P2X2 subcloned into pcDNA3, wild-type GluA1–4 subunits or super-ecliptic-pHluorin (SEP)-tagged GluA1 or GluA2 subunits subcloned into PrK5 vector have been described previously^10^. Single or double mutations of GluA1 or GluA3 subunits were generated sequentially using the QuikChange site directed mutagenesis method with specific oligonucleotides corresponding to each mutation (Stratagene). GluA1CTA2, GluA1CTA3 chimeras were generated using restriction site addition by PCR and subcloned into pcDNA3. The N-terminal domain of GluA1 was amplified by PCR using pfu polymerase (Fermentas) and primers in order to create 5′ HindIII and 3′ EcoRI restriction site at the junction between TM3 and CT. The EcoRI site naturally present at the same position on GluA2 or GluA3 was used to fuse in-frame the C-term of GluA2 or GluA3 to the N-term of GluA1. The additional EcoRI site present in the CT of GluA2 was first removed by silent mutation using the QuikChange method. Conversely, GluA3CTA1 chimeras were generated by exchange of the C-terminal sequence using the natural EcoRI restriction site on GluA3 and one created by PCR into the same sequence on GluA1. GluA1loop1A3 and Glu3loop1A3 were generated by substitution of the first intracellular loop 1 domain of one GluA subunit by the other one using the Quickchange method and megaprimers. Megaprimers were generated by PCR: for example, the loop1 of GluA3 was first amplified from GluA3 by PCR using primers with flanking regions corresponding to adjacent GluA1 sequences. The PCR product was then used as megaprimers on GluA1 to generate GluA1loop1A3 by the Quikchange method. All constructs were verified by sequencing.

### Xenopus oocyte electrophysiology

Oocytes were removed from *Xenopus laevis* as previously described^34^,^35^. After nuclear injection of cDNAs coding for each wild-type or modified AMPAR subunits (each 50–80 pg) alone or with P2X2 (10–30 pg), oocytes were incubated in Barth’s solution containing 1.8 mM CaCl_2_ and gentamycin (10 mg/ml, Sigma) at 19 °C for 1–3 days before electrophysiological recordings were performed as previously described^10^. Two-electrode voltage-clamp recordings were conducted at room temperature using glass pipettes (tip resistance 1–2 MΩ) filled with 3 M KCl solution to ensure a reliable holding potential. Oocytes were voltage-clamped at −60 mV, and membrane currents were recorded with an OC-725B amplifier (Warner Instruments) and digitized at 1 kHz on a Power PC Macintosh G4 using Axograph X software (Axograph). Oocytes were superfused at a flow rate of 10-12 ml/min with Ringer solution, pH 7.4, containing in mM: 115 NaCl, 3 NaOH, 2 KCl, 1.8 CaCl_2_, and 10 HEPES. Agonists and drugs were prepared at their final concentrations in the perfusion solution and applied using a computer- driven valve system (Ala Scientific).

### Hippocampal neuron culture and transfection

Primary hippocampal cultures were prepared from E18 Sprague-Dawley rat embryos according to the Banker protocol (Kaech and Banker, 2006). Hippocampi were dissected in Petri dishes filled with HBSS and HEPES, and dissociated by trypsin treatment (0.05%; Gibco) at 37 °C followed by trituration with flame-polished Pasteur pipettes. Cells were electroporated and plated at a density of 250,000 per 60-mm dish, on poly-L-lysine pre-coated 1.5H coverslips (Marienfield, cat. No. 117580), pre-plated with 75,000 non-electroporated cells. After 2 hours, coverslips were transferred to dishes containing an astrocyte feeder layer, plated at a density of 40,000 cells and cultured in MEM (Fisher scientific, cat No. 21090-022) containing 4.5 g/l Glucose, 2 mM L-glutamine and 10% horse serum (Invitrogen) for 14 days. Neuron cultures were maintained in Neurobasal medium supplemented with 2 mM L-glutamine and 1X NeuroCult SM1 Neuronal supplement (STEMCELL technologies) for 14–16 days.

### Direct Stochastic Optical Reconstruction Microscopy

Neuronal cultures of 14–16 DIV electroporated with either wildtype or mutant SEP-tagged-GluA1. ATP treatments were conducted in the presence of tetrodotoxin (TTX 0.5 μM) and the adenosine receptor antagonist CGS15943 (3 μM), were incubated with anti-GFP antibodies (1:1000, Roche) in culture medium at 37 °C for 6 minutes and fixed in 4% paraformaldehyde and sucrose in PBS for 10 minutes. After three washes in PBS, they were incubated with NH_4_Cl 50 mM for 10 minutes. After three washes in PBS, they were again incubated with PBS containing 2% Bovin Serum Albumin (BSA) (Sigma-Aldrich, St. Louis, MO) for 45 minutes. The Primary antibodies were then revealed by incubating with Alexa647-coupled donkey anti-mouse IgG secondary antibodies (1:500, Jackson lab) for 45 minutes at room temperature. After three washes in PBS containing 2% BSA and 3 washes in PBS, coverslips were again fixed using a previously described protocol and kept in PBS.

The stained coverslips were imaged during the next week at room temperature in a closed chamber (Ludin Chamber, Life Imaging Services, Switzerland) mounted on a Leica SR GSD microscope (Leica Microsystems, Wetzlar, Germany) equipped with a 160 × 1.47 NA objective and an iXon3 EMCCD camera (ANDOR, Belfast, UK). Imaging was performed in an extracellular solution containing a reducing and oxygen scavenging system. For direct Stochastic Optical Reconstruction Microscopy, ensemble fluorescence of Alexa647 were first converted into a dark state using a 642 nm laser 30–50 kw/cm^2^. Once the ensemble fluorescence was converted into a desired number of single molecules per frame, the laser power was reduced to 7–15 kw/cm^2^ and imaged continuously at 50fps for 90,000 frames. Both the ensemble and single molecule fluorescence was collected by a combination of dichroic and emission filters (D101-R561 and F39-617, respectively, Chroma, USA) and a quad-band dichroic filter (Di01-R405/488/561/635, Semrock, USA). Super-resolution images were reconstructed using a Leica GSD analysis program and corrected for lateral drift using multicolor fluorescent microbeads (Tetraspeck, Invitrogen). The AMPAR density determination was done in two steps. The first consisted of determining the intensity of isolated single particles. The second step was to divide the dendritic and synaptic intensities by the single particle intensity^20^.

### Hippocampal slice preparation

Horizontal hippocampal slices were prepared from 4–5 -week-old C57BL6 mice as previously described^10^. Animals were anesthetized with isoflurane gas and then decapitated. Brains were rapidly removed and immersed in ice-cold artificial cerebrospinal fluid (ACSF) containing 125 mM NaCl, 2.5 mM KCl, 1.25 mM NaH_2_PO_4_, 26 mM NaHCO_3_, 2 mM CaCl_2_, 1.3 mM MgCl_2_ and 25 mM glucose saturated with 95% O_2_/5% CO_2_^36^. Horizontal hippocampus slices (350 μm thick) were obtained from brains using a vibratome (Leica VT 1200 s). The slices were transferred to an interface storage chamber containing ACSF saturated with 95% O_2_/5% CO_2_ and were left at least 45 min at 35 °C to recover and then were maintained at room temperature for 45 min. Slices were placed into ACSF containing picrotoxin (100 μM, Santa Cruz), D-AP5 (10 μM, Santa Cruz) and CGS 15943 (3 μM, Santa Cruz) to block GABA_A_, NMDA and adenosine receptors, respectively. For NMDAR-dependent-LTD experiments, D-AP5 was omitted from the bathing ACSF. P2X- or NMDA induced LTD was induced by submerging slices in ATP (300 μM) for 10 min or NMDA (20 μM) for 3 min^10^,^16^. After agonist treatment, slices were rinsed with ACSF, transferred to another well containing ACSF for 5 or 30 min then quickly placed in cold PBS. Hippocampi were immediately dissected out and placed at −80 °C. Control slices were manipulated in the exact same way but were not subjected to agonist treatment.

### Western blotting analysis

Homogenates of two hippocampus slices were prepared by sonicating on ice during 10 s in 10 μl of a homogenization buffer per slice containing a cocktail of 0.1 M Tris pH 8.0, 10 mM EDTA, Halt protease and phosphatase inhibitors (Thermo Scientific) and 1 μM okadaïc acid (Santa Cruz). The protein concentration was determined by the BCA method (Pierce) and 20 μg of proteins per lane were loaded onto an 8% SDS-PAGE gel and then transferred to polyvinylidene difluoride (PVDF) membranes. Western blotting using phospho-S567 revealed no or at most a faint band arising from total proteins (see Supplementary Fig. 2). To quantify the phospho-Ser 567 signals, homogenates were first immunoprecipated using anti-GluA1 antibodies as previously described^18^. 100 μg of homogenates were incubated with 2 μg of anti-GluA1 antibodies (Alomone) and 20 μl of protein G-dynabeads (Pierce) at 4 °C overnight. Beads were washed four times with Homogenization buffer and eluted in an SDS sample buffer. Membranes were saturated for 60 min by incubation with 5% Bovin Serum Albumin (BSA) in a PBS (2 mM KH_2_PO_4_, 150 mM NaCl, 8 mM NaH_2_PO_4_.2H_2_O) containing 0.1% Tween 20 and incubated overnight with the following antibodies: anti-pS831 (1/500, Millipore), anti-pS845 (1/1000, Millipore) or anti p-S567 (1/500, kindly provided by Katherine Roche), anti-actin (1/10,000, Sigma) or anti-GluA1 (1/200, Alomone). Membranes were then incubated for 1 h at room temperature with a secondary anti-mouse or anti-rabbit horseradish peroxidase-coupled antibody (both at 1:5000; Jackson ImmunoResearch) diluted in PBS-Tween 0.1% and supplemented with 5% non-fat milk. Signals were revealed by chemiluminescence (Millipore) and images were acquired on a Chemidoc System (Bio-rad). Quantification of western blots was performed using Image J software (National Institutes of Health), whereby phospho-specific/GluA1 or phospho-specific/actin ratios were determined. (For full length blots see Supplementary Fig. 1).

### Statistics

Statistical analysis was performed using the Student’s t test, or ANOVA Kruskal-Wallis test with Dunn’s *post-hoc* procedure for between-group comparisons (Prism 6.0, Graphpad). Data were considered significantly different when the P value was less than 0.05. Data in Figs 1, 2, 3, 4 were also compared to the null hypothesis of the zero inhibition group to determine whether the degree of inhibition (expressed as %) was reduced or abolished (non significantly different from 0% of inhibition). All statistical results are given as the mean ± s.e.m.

### Study approval

All experiments were carried out in accordance with the European Communities Council Directive and approval by the ethics committee of the University of Bordeaux (CEEA50).

## Additional Information

**How to cite this article**: Pougnet, J.-T. *et al.* P2X-mediated AMPA receptor internalization and synaptic depression is controlled by two CaMKII phosphorylation sites on GluA1 in hippocampal neurons. *Sci. Rep.* **6**, 31836; doi: 10.1038/srep31836 (2016).

## Supplementary Material

Supplementary Information
