# Supplementary material for: P2X-mediated AMPA receptor internalization and synaptic depression is controlled by two CaMKII phosphorylation sites on GluA1 in hippocampal neurons
Source: Sci Rep. 2016 Sep 14;6:31836. doi: 10.1038/srep31836 (PMC5022001; doi:10.1038/srep31836)
Supplement: Supplementary Information [file srep31836-s1.pdf]

Supplementary Information

P2X-mediated AMPA receptor internalization and synaptic depression is controlled by two CaMKII phosphorylation sites on GluA1 in hippocampal neurons

Johan-Till Pougnet[1,2], Benjamin Compans[3,4], Audrey Martinez[1,2], Daniel Choquet[3,4,5], Eric Hosy[3,4] and Eric Boué-Grabot*[1,2]

[1] Univ. de Bordeaux, Institut des Maladies Neurodégénératives, UMR 5293, F-33000 Bordeaux, France;

[2] CNRS, Institut des Maladies Neurodégénératives, UMR 5293, F-33000 Bordeaux, France;

[3] Univ. de Bordeaux, Institut Interdisciplinaire des Neurosciences, UMR 5297, F-33000 Bordeaux, France;

[4] CNRS, Institut Interdisciplinaire des Neurosciences, UMR 5297, F-33000 Bordeaux, France.

[5] Bordeaux Imaging Center, UMS 3420-US4 CNRS, INSERM, Université de Bordeaux

*: Address correspondence to:

Eric Boué-Grabot, Ph.D.
Institut des Maladies Neurodégénératives (IMN)
CNRS UMR 5293,
Université de Bordeaux,
146 rue Léo Saignat,
33076 Bordeaux cedex,
France.
Tel: +33 5 57575763;
Fax: +33 5 57575760;
E-mail: eric.boue-grabot@u-bordeaux.fr

Supplementary Figure 1

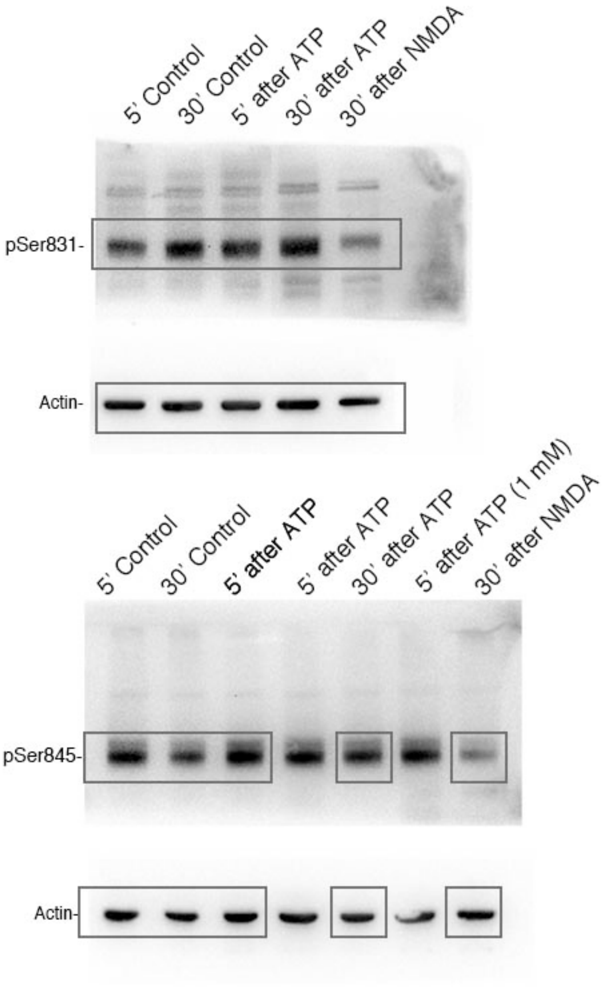

Fig. 6B

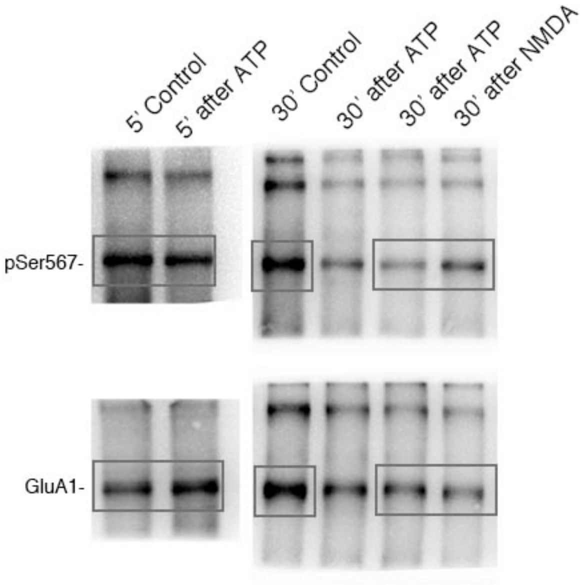

Fig. 6C

Supplementary figure 1. Full length version of western blots showed in Fig.6B and C.

Lane of blots used to create Fig 6B and 6C panels are framed.

Supplementary Figure 2

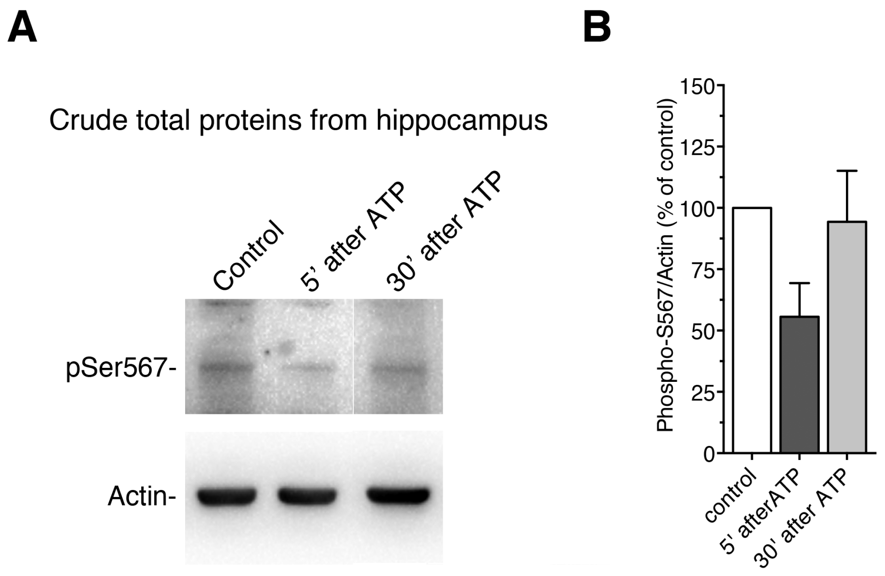

Supplementary Figure 2. (A) Western blot using anti-phospho-S567 and actin **from** crude membrane fractions from control hippocampal slices and ATP-induced synaptic depression slices taken at indicated times 5' or 30' after the application of ATP (300 μM for 10 min). In these conditions, the detection of Phospho-S567 revealed no (5 experiments) or at most a faint band (n=2 independent experiments) arising from total proteins difficult to accurately quantify. We thus conducted immunoprecipitation using anti-GluA1 antibodies prior p-S567 detection (see Fig. 6C). (**B)** Quantification of the relative amounts of phosphorylation of GluA1-S567 detected from crude membranes (n=2) showed a transient dephosphorylation of the S567 site of the AMPAR after P2X activation from hippocampal slices, in agreement with results obtained after immunoprecipitation (Fig. 6C).
